# Artificial Intelligence–Driven Respiratory Distress Syndrome Prediction for Very Low Birth Weight Infants: Korean Multicenter Prospective Cohort Study

**DOI:** 10.2196/47612

**Published:** 2023-07-10

**Authors:** Woocheol Jang, Yong Sung Choi, Ji Yoo Kim, Dong Keon Yon, Young Joo Lee, Sung-Hoon Chung, Chae Young Kim, Seung Geun Yeo, Jinseok Lee

**Affiliations:** 1 Biomedical Engineering Kyung Hee University Yongin-si Republic of Korea; 2 Department of Pediatrics Kyung Hee University Medical Center Kyung Hee University College of Medicine Seoul Republic of Korea; 3 Department of Obstetrics and Gynecology Kyung Hee University Medical Center Kyung Hee University College of Medicine Seoul Republic of Korea; 4 Department of Pediatrics Kyung Hee University Hospital at Gangdong Kyung Hee University College of Medicine Seoul Republic of Korea; 5 Department of Otorhinolaryngology Head and Neck Surgery Kyung Hee University Medical Center Kyung Hee University School of Medicine Seoul Republic of Korea

**Keywords:** artificial intelligence, deep neural network, premature infants, respiratory distress syndrome, AI, AI model, pediatrics, neonatal, maternal health, machine learning, deep neural network

## Abstract

**Background:**

Respiratory distress syndrome (RDS) is a disease that commonly affects premature infants whose lungs are not fully developed. RDS results from a lack of surfactant in the lungs. The more premature the infant is, the greater is the likelihood of having RDS. However, even though not all premature infants have RDS, preemptive treatment with artificial pulmonary surfactant is administered in most cases.

**Objective:**

We aimed to develop an artificial intelligence model to predict RDS in premature infants to avoid unnecessary treatment.

**Methods:**

In this study, 13,087 very low birth weight infants who were newborns weighing less than 1500 grams were assessed in 76 hospitals of the Korean Neonatal Network. To predict RDS in very low birth weight infants, we used basic infant information, maternity history, pregnancy/birth process, family history, resuscitation procedure, and test results at birth such as blood gas analysis and Apgar score. The prediction performances of 7 different machine learning models were compared, and a 5-layer deep neural network was proposed in order to enhance the prediction performance from the selected features. An ensemble approach combining multiple models from the 5-fold cross-validation was subsequently developed.

**Results:**

Our proposed ensemble 5-layer deep neural network consisting of the top 20 features provided high sensitivity (83.03%), specificity (87.50%), accuracy (84.07%), balanced accuracy (85.26%), and area under the curve (0.9187). Based on the model that we developed, a public web application that enables easy access for the prediction of RDS in premature infants was deployed.

**Conclusions:**

Our artificial intelligence model may be useful for preparations for neonatal resuscitation, particularly in cases involving the delivery of very low birth weight infants, as it can aid in predicting the likelihood of RDS and inform decisions regarding the administration of surfactant.

## Introduction

Respiratory distress syndrome (RDS), a disease that commonly affects premature infants, results from insufficient synthesis of the pulmonary surfactant in the alveoli [1]. The more premature the infants are, the greater is the likelihood that they will be affected with RDS; thus, this disease is fundamental in the setting of premature infants [2]. Patients with RDS can be effectively treated with artificial pulmonary surfactant, which prevents collapse of the air sacs of the lungs by reducing surface tension [3]. However, a recent study reported that use of a less invasive surfactant administration (LISA) technique can reduce the need for mechanical ventilation in infants with RDS and reduce the composite outcome of death or bronchopulmonary dysplasia [4,5]. Despite the usefulness of LISA, preemptive treatment with artificial pulmonary surfactant is currently administered to most premature infants, regardless of RDS diagnosis [6]. However, synthetic surfactant treatment of established RDS may lead to an increase in apnea of prematurity [7,8]. Thus, because artificial pulmonary surfactant can only be administered to infants with RDS who require treatment, accurate prediction of RDS in premature infants at birth is essential.

The clinical guidelines for the prediction or detection of RDS are complicated. The diagnosis of RDS is based on a combination of clinical signs, symptoms, chest radiographic findings, and arterial blood gas results [1]. Recently, artificial intelligence (AI) technologies have been addressing these difficult issues in diagnosis or prediction that arise from complex causal relationships [9-11]. Machine learning models based on large-scale patient data have recently been introduced for the prediction of RDS [12-16]. Although these machine learning models showed accurate prediction performance, most of the data were obtained from adult patients. Only a few studies have used machine learning models for the prediction of RDS in premature infants [17-19]. A study involving training and evaluation for a logistic regression (LR) model for late-preterm and full-term infants has been reported [17]; the area under receiver operating characteristic (AUROC) curve in that study was 0.760. Although full-term infants were evaluated in that study [17], the model accuracy was low. Another study [18] reported on the training and evaluation of a gradient boosting machine (GBM) for infants born prior to 39 weeks in gestational age; the AUROC was 0.923, demonstrating the strong potential of machine learning models in the prediction of RDS. However, that study [18] consisted of term and preterm infants for whom AI-driven models were suggested for RDS diagnosis. Considering that RDS is mainly a preterm birth–based disease, this heterologous and skewed data set consisting of term infants caused a large bias in the AI-driven model. Thus, the results were inconclusive due to small sample sizes and the general population-skewed data set. Another study [19] reported on the training and evaluation of support vector machine (SVM), random forest (RF), and artificial neural network. The AUROC of these machine learning models was also high, ranging from 0.97 to 1.00. However, those results were also inconclusive due to small sample sizes, and the study did not consider very low birth weight infants (VLBWIs).

In this study, research on AI, including machine learning models, was extended by the use of a nationwide large-scale data set based on the registry network of VLBWIs in South Korea [20]. The database known as the Korean Neonatal Network (KNN) was established in April 2013, with enrollment of 2000 to 3000 VLBWIs (less than 1500 grams) every year. As of October 2022, the number of cumulative patients was approximately 18,246 [20,21]. The 76 member hospitals of KNN in South Korea have a data management board for the maintenance of qualified nationwide data on VLBWIs [22].

Using the large-scale data set, we performed a comparison of the prediction performances of 7 different machine learning models, namely, GBM, LR, extreme gradient boosting (XGBoost), adaptive boosting (AdaBoost), light GBM (LGBM), RF, and SVM. The best classifier was then selected, and an analysis of feature importance was performed in order to assess the contribution of each feature. A 5-layer deep neural network (DNN) with the selected features derived from the best machine learning classifier was then proposed in order to enhance the prediction performance. The most significant contributions of this study are as follows:

1. Development and validation of AI models for the prediction of RDS was based on the large amount of data on Korean VLBWIs (13,087 newborn infants weighing less than 1500 grams).
2. The prediction performances of 7 different machine learning models, namely, GBM, LR, XGBoost, AdaBoost, LGBM, RF, and SVM were compared.
3. The best classifier was selected and an analysis of feature importance was performed in order to assess the contribution of each feature.
4. A 5-layer DNN model based on the selected features derived from the best machine learning classifier was proposed in order to enhance the prediction performance.
5. Finally, a public web application was deployed that enables easy access and use of our proposed AI models for RDS prediction [23].

## Methods

### Ethics Approval

The national KNN registry was approved by the institutional review boards of all 76 hospitals participating in the KNN (2022-ER0603-01#) [24]. In addition, the protocol for this study was approved by the institutional review board of Kyung Hee University Hospital, Seoul, Korea (KHUH2013-03-103). Written consent was obtained from the parents or legal guardians of the infants during enrollment in the KNN. All procedures were performed in accordance with the relevant guidelines and regulations.

### Data for the AI Model

The training and evaluation of the AI models for the prediction of RDS were based on a prospectively built registry of newborn infants weighing less than 1500 grams in the KNN between 2013 and 2021. The KNN included 14,519 VLBWIs; of these, 1399 sets of data were missing and data for infants older than 33 weeks were not used due to the small sample size. Therefore, data on 13,087 VLBWIs were used in this study. Data regarding each infant were collected under 6 categories of information: basic information regarding the infants, maternity, pregnancy/birth process, family, resuscitation, and test results at birth. In particular, basic information on the infants included sex (male/female), birth weight (grams), birth height (cm), birth head circumference (cm), birthplace (in hospital, transferred from another hospital after birth, or not in hospital), and body temperature (°C) at the initial admission. Information regarding maternity history included maternal age (years), diabetes (yes/no), hypertension (yes/no), and gravida (number). Information regarding the pregnancy/birth process included gestational age (weeks), amount of amniotic fluid (normal, oligohydramnios, or hydramnios), premature rupture membrane (yes/no), antenatal steroid (yes/no), delivery types (caesarean section or natural birth), and in vitro fertilization (yes/no). Information regarding the family history included parity defined as the number of times that she has given birth to a fetus with a gestational age of 24 weeks or more, marriage (married, divorced, single, or unmarried cohabitation), multiple gestation (singleton, twin, triplet, or more), and birth order (first-, second-, third-order, or more). Information regarding resuscitation procedure included resuscitation at birth (yes/no) as well as the treatment types such as oxygen, positive pressure ventilation, tracheal intubation, cardiac compression, and medical usage. The test results at birth included 1-minute Apgar score, 5-minute Apgar score, blood gas analysis, and blood gas analysis base excess. Regarding the outcomes (RDS or non-RDS), a diagnosis of RDS was made by neonatologists according to the clinical features and chest radiographic findings. A statistical summary of 30 clinical variables according to RDS and non-RDS is shown in Table 1. *P* values were based on a 2-sided *t* test for means and a chi-square test for categorical variables [25,26]. A 2-sided *P* value <.05 was considered significant.

**Table 1:** Statistical summary of the clinical variables in the respiratory distress syndrome group and non–respiratory distress syndrome group.

Data				RDS^a^ group (n=10,041)		Non-RDS group (n=3046)		*P* value^b^	
**Basic infant information**									
	**Gender, n**								>.99
		Male	5165		1395				
		Female	4876		1651				
	Birth weight (g), mean (SD)			1022.01 (284.26)		1269.24 (198.43)		<.001	
	Birth height (cm), mean (SD)			35.81 (3.64)		38.55 (2.75)		.45	
	Birth head circumference (cm), mean (SD)			25.37 (2.44)		27.62 (1.74)		<.001	
	**Birthplace, n**								.84
		In hospital	9871		3009				
		Transferred from another hospital after birth	161		34				
		Not in hospital	9		3				
**Maternity history, mean (SD)**									
	Body temperature at the initial admission (°C)			36.14 (0.63)		36.30 (0.48)		<.001	
	Maternal age (years)			33.27 (4.29)		33.20 (4.09)		.78	
	**Diabetes, n**								.005
		No diabetes	9041		2761				
		Gestational diabetes mellitus	857		255				
		Overdiabetes mellitus	143		30				
	**Hypertension, n**								<.001
		No hypertension	8083		2090				
		Pregnancy-induced hypertension	1718		896				
		Chronic hypertension	240		60				
	Gravida, mean (SD)			1.98 (1.22)		1.84 (1.14)		<.001	
**Pregnancy/birth process**									
	Gestational age (weeks), mean (SD)			27.56 (2.53)		31.54 (2.36)		<.001	
	**Amount of amniotic fluid, n**								.93
		Normal	8415		2553				
		Oligohydramnios	1452		492				
		Hydramnios	174		30				
	Premature rupture of membrane, n			3713		789		.38	
	Antenatal steroid, n			8764		2567		.77	
	Delivery type (caesarean section), n			7950		2510		<.001	
	In vitro fertilization, n			2455		769		.07	
**Family history**									
	Parity, mean (SD)			0.49 (0.73)		0.40 (0.67)		<.001	
	**Marriage, n**								.07
		Married	9862		3006				
		Divorced	6		3				
		Single	62		21				
		Unmarried cohabitation	111		16				
	**Multiple gestation, n**								<.001
		Singleton	6534		1756				
		Twin	3168		1070				
		Triplet	332		207				
		Quadruplet or more	7		13				
	**Birth order of multiple gestation, n**								<.001
		First order	1585		505				
		Second order	3612		1406				
		Third order	366		231				
		Fourth order or more	4		20				
**Resuscitation procedure, n**									
	Resuscitation at birth			9634		2029		<.001	
	Oxygen usage			8854		1897		.42	
	Positive pressure ventilation usage			8830		1323		<.001	
	Tracheal intubation			7384		391		.02	
	Cardiac compression			523		32		.02	
	Medication usage			386		23		.32	
**Test results at birth, mean (SD)**									
	1-min Apgar score			4.32 (1.97)		6.14 (1.79)		<.001	
	5-min Apgar score			6.56 (1.86)		8.02 (1.38)		<.001	
	Blood gas analysis (pH)			7.26 (0.12)		7.27 (0.09)		<.001	
	Blood gas analysis base excess			–5.39 (4.40)		–4.30 (3.41)		<.001	

^a^RDS: respiratory distress syndrome.

^b^*P* values are based on the 2-sided *t* test for means and a chi-square test for categorical variables. A 2-sided *P* value <.05 was considered significant.

### Preprocessing, Data Split, Data Balancing, and Cross-Validation

Among the 30 variables summarized in Table 1, we applied one-hot encoding to 6 categorical variables, that is, the amount of amniotic fluid, marriage, multiple gestation, diabetes, hypertension, and birthplace. One-hot encoding is used for variables where order does not matter, and it creates dummy variables. Each dummy variable has a value of 0 or 1. For the amount of amniotic fluid variable, we created 3 dummy variables, namely, normal, oligohydramnios, and hydramnios. For the marriage variable, we created 4 dummy variables, namely, marriage, divorced, single, and unmarried cohabitation. For the multiple gestation, we created 4 dummy variables, namely, singleton, twin, triplet, and quadruplet. In a similar way, diabetes was assigned with 3 dummy variables, namely, no diabetes, gestational diabetes mellitus, and overdiabetes mellitus. Hypertension was assigned with 3 dummy variables, namely, no hypertension, pregnancy-induced hypertension, and chronic hypertension. Birthplace was assigned with 3 dummy variables, namely, in-hospital, transferred from another hospital after birth, and not in a hospital.

A total of 47 inputs or features were then applied for the AI model inputs. For training and testing the model, data on 13,087 infants were divided into training (n=10,469) and testing data (n=2618) in a ratio of 8:2 in a stratified manner. Then, training and testing data include the same ratio of RDS (training data, 8032/10,469, 76.72%; testing data, 2009/2618, 76.72%) and non-RDS (training data, 2437/10,469, 23.28%; testing data, 609/2618, 23.28%). A summary of the data sets for training and testing is shown in Table 2. We used the testing set for performance evaluation only as independent data of the AI model that we developed [27]. For training the model, a 5-fold cross-validation was performed using the training data in order to confirm the generalization ability of the model. First, random shuffling of the training data was performed; it was then divided into 5 equal groups in a stratified manner. Each of 5 groups also includes the same ratio of RDS and non-RDS. One group was subsequently used for internal validation, and other 4 groups were used for internal validation. We repeated this process by shifting the internal validation group. In addition, due to a much higher amount of RDS data (training data, 8032/10,469, 76.72%; testing data, 2009/2618, 76.72%) compared with non-RDS data (training data, 2437/10,469, 23.28%; testing data, 609/2618, 23.28%), the numbers of data from the 2 classes of RDS and non-RDS were balanced. In order to balance the classes, upsampling of the non-RDS data was performed using the Synthetic Minority Over-sampling Technique with Tomek links during update of the model [28]. We were able to minimize the model bias, particularly toward a majority of groups, by balancing the 2 classes.

**Table 2:** Summary of the training and testing data sets.

	RDS^a^ (n=10,041), n	Non-RDS (n=3046), n
Training data (n=10,469)	8032	2437
Testing data (n=2618)	2009	609

^a^RDS: respiratory distress syndrome.

### Machine Learning Models

**Figure 1:**
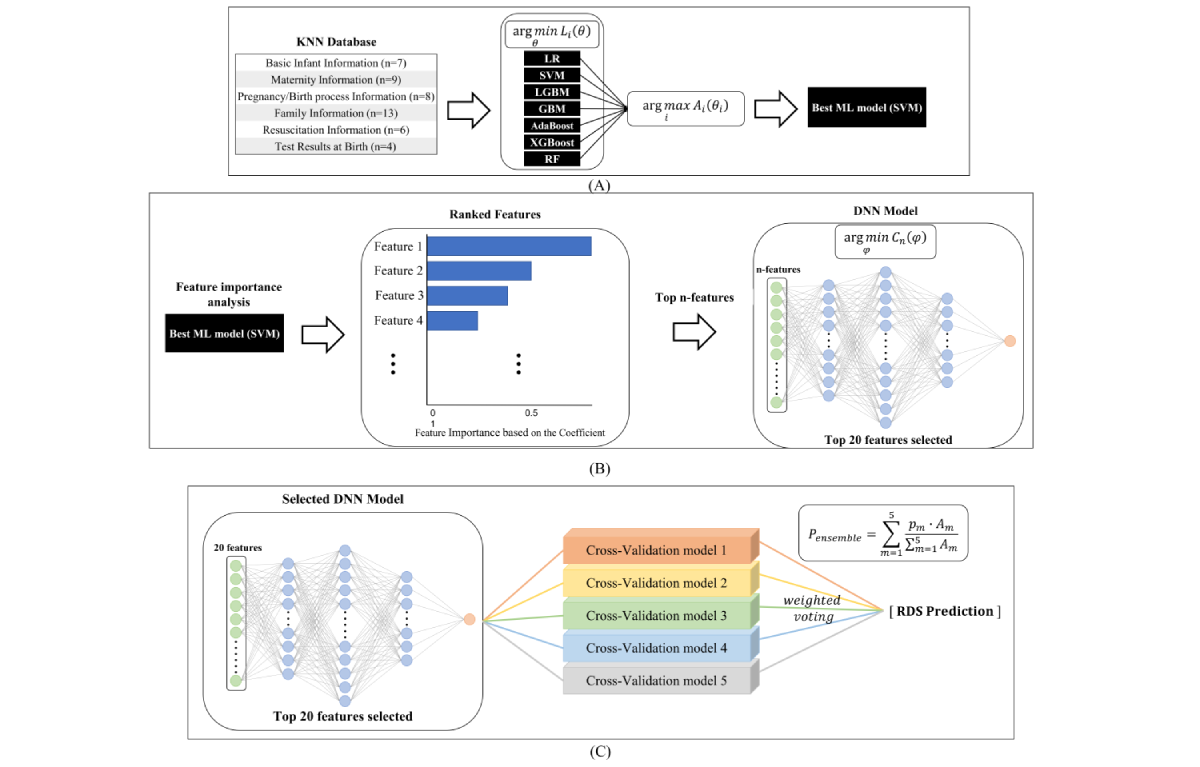
Ensemble approach for the classification of testing data. AdaBoost: adaptive boosting; DNN: deep neural network; GBM: gradient boosting machine; KNN: Korean neonatal network; LGBM: light gradient boosting machine; LR: logistic regression; ML: machine learning; RDS: respiratory distress syndrome; RF: random forest; SVM: support vector machine; XGBoost: extreme gradient boosting.

An illustration of our proposed overall network architecture for training and testing data is shown in Figure 1. First, preprocessing of the 47 features was performed for normalization of data. The preprocessed data were then fed into 7 different machine learning models: XGBoost [29,30], AdaBoost [31,32], GBM [33,34], LGBM [35], RF [36], LR [37], and SVM [38]. As illustrated in Figure 1A, for each model, the objective is to identify the optimized hyperparameters as follows:

argmin_θ_ [*L_i_(θ)*] **(1)**

where *i* represents each of 7 models, *L_i_(∙)* represents the cost function for each model *i*, and θ represents the hyperparameter set. More specifically, the optimized hyperparameters *θ_i_* for each model *i* were determined using 5-fold cross-validation. Two evaluation indicators were used in the validation to determine the optimized hyperparameters: balanced accuracy and AUROC from internal validation data. The balanced accuracy was calculated by averaging sensitivity and specificity. The AUROC was calculated by finding the area of ROC curves. In particular, considering the imbalance between the RDS and non-RDS group, balanced accuracy was used as the main evaluation indicator. Note that the use of Synthetic Minority Over-sampling Technique with Tomek links was not applied to internal validation data and testing data. After determining the optimized hyperparameters *θ_i_* for each model *i,* the best model was selected as follows:

argmax_i_ [*A_i_(θ_i_)*] **(2)**

where *A_i_(θ_i_)* represents the balanced accuracy of internal validation data from each model *i*.

### Feature Importance Analysis

An analysis of feature importance was performed using the best model from the machine learning models, which ranks the features in the order of importance. For a decision tree approach such as RF, XGBoost, GBM, LGBM, and AdaBoost, the feature importance values were calculated as the decrease in node impurity weighted by the probability of reaching the node. The node probability was calculated as the number of samples reaching the node divided by the total number of samples. The node impurity was based on the Gini index, which measures the degree of a particular variable being incorrectly classified when it is randomly chosen. However, for a coefficient approach such as SVM and LR, the feature importance values were calculated by the absolute value of the coefficients or weights. The values measure how much a marginal change in the feature value would affect the outcomes.

### DNN Based on Top n-Features With Feature Selection

The optimized DNN models were determined for each set of top *n*-features, as illustrated in Figure 1B. For the DNN model inputs, we first ranked the features based on the resultant feature importance values from the best classifier by using machine learning models. Then, we used top *n*-features as the model input, where *n* was changed from 1 to 47: *n* represents the model input set from top 1 feature to top 47 features. For each DNN model, we identified the optimized hyperparameters such as layer depth and width as

argmin_φ_ [*C_n_(φ)*] **(3)**

where φ represents the hyperparameter set. All combinations of layer depth and width were considered by changing the number of hidden layers from 1 to 5 and the number of nodes (width) from 1 to the number of previous layer nodes. Cn(φ) is the cross-entropy cost of binary classification defined by 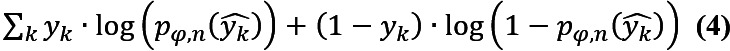, where *y_k_* denotes the true label (0 or 1), 
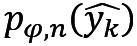
 denotes the softmax probability corresponding to *y_k_* for top *n*-feature input given the optimized hyper-parameter set *φ*.

The best cross-validation accuracy was determined for each set of top *n*-features by using the 2 metrics of balanced accuracy and AUROC. The 2 metrics were used to evaluate each model performance because of the imbalanced classes between the RDS and non-RDS group. As a result, an optimized 5-layer DNN was identified using the top 20 features as the best model for the prediction of RDS. We have shown the effects of the number of selected top *n*-features (1 to 47) in the Results section. The 5-layer DNN consisted of an input layer for the top 20 features, 3 fully connected layers, and an output layer. The 3 fully connected layers consisted of 32, 64, and 16 nodes. The last fully connected layer was fed into a sigmoid layer for predicting the probability of RDS. Batch normalization and dropout with a dropout rate of 0.5 were applied before each of the fully connected layers in our proposed DNN model. An adaptive moment estimation optimizer with a batch size of 32 and binary cross-entropy loss function with a learning rate of 0.0001 on an NVIDIA GeForce GTX 1080 Ti GPU were used for training the models. For the analysis, training, and evaluation of machine learning and deep learning models, we used the following packages: Python (version 3.9) with TensorFlow (version 2.8.0), Pandas (version 1.3.4), Matplotlib (version 3.4.3), Keras (version 2.8.0), NumPy (version 1.20.3), and Scikit-learn (version 1.0.2).

### Final AI Model: Ensemble DNN for Testing

An ensemble approach combining the 5 models derived from the 5-fold cross-validation was adopted for the final DNN-based AI model. An illustration of the ensemble model is shown in Figure 1C. The average of balanced accuracies can be denoted from the internal validation data set in the *m*-th cross-validation model by *A_m_* where *m*={1, 2, …, 5}. In addition, the estimated probability of RDS can be denoted from the testing data set in the *m*-th cross-validation model by *p_m_*. The weighted probability was then calculated as 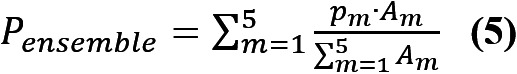, where *P_ensemble_* denotes the final probability in an ensemble approach by applying the weight value of *A_m_* to the probability value of *p_m_* Thus, 5 probabilities were weighted according to the average values for balanced accuracy, which is a weighted soft voting. Evaluation of the prediction performance based on the ensemble DNN model was performed using the independent testing data set (n=2618).

### AI-Driven Web Application

Our proposed ensemble DNN model including the top 20 features was deployed on our own public website [23], so that RDS can be predicted if information on the selected 20 features from VLBWI is available. After accessing the website, information regarding the features is entered by the user and then encoded to the website server for immediate prediction of RDS. Other than the information regarding features, there is no need for entering any information that would be regarded as private, and the information that is entered is immediately deleted upon generation of the prediction result, so that there is no risk of exposing information. The code is also available at [39].

## Results

### Feature Importance Analysis

**Figure 2:**
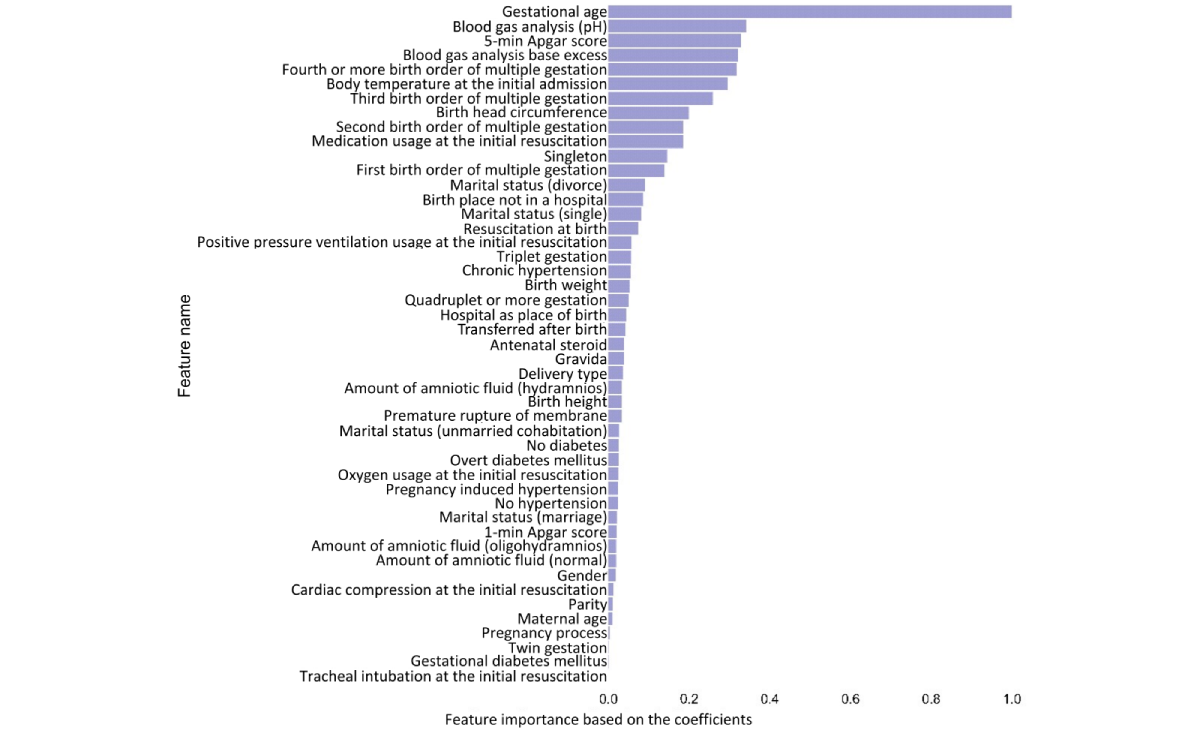
Results of feature importance analysis by support vector machine.

A summary of the results of cross-validation from 7 different machine learning algorithms, that is, XGBoost, AdaBoost, GBM, LGBM, RF, LR, and SVM, is shown in Table 3. According to the results, the SVM model was the best classifier for the prediction of RDS in newborn VLBWIs weighing less than 1500 grams. The accuracy value for the SVM model was 0.84, with a balanced accuracy of 0.84, and an AUROC of 0.92, which were higher than those for other machine learning models. Next, an analysis of feature importance was performed using the best model, that is, SVM. The results showing the ranked feature importance using SVM indicated that gestational age showed the highest importance value, followed by blood gas analysis, 5-minute Apgar score, blood gas analysis base excess, and the fourth or more birth order of multiple gestation (Figure 2). These results are consistent with findings from recent studies demonstrating the usefulness of gestational age, results of blood gas analysis, and Apgar score in the early detection of RDS [40,41]. The results of feature importance analysis from other 6 machine learning models are shown in Figures S1-S6 of Multimedia Appendix 1. Compared to SVM, the other 6 models were observed to be biased toward specific features.

**Table 3:** Mean (SD) data of the cross-validation results.

Model	Sensitivity	Specificity	Accuracy	Balanced accuracy	AUROC^a^
XGBoost^b^	0.8662 (0.006)	0.7655 (0.014)	0.8498 (0.004)	0.8309 (0.006)	0.9061 (0.005)
AdaBoost^c^	0.8546 (0.005)	0.8206 (0.012)	0.8467 (0.005)	0.8377 (0.006)	0.9076 (0.006)
GBM^d^	0.9110 (0.005)	0.7215 (0.001)	0.8669 (0.002)	0.8162 (0.004)	0.9114 (0.002)
LGBM^e^	0.8927 (0.007)	0.7462 (0.014)	0.8587 (0.002)	0.8194 (0.003)	0.083 (0.004)
RF^f^	0.8992 (0.006)	0.7490 (0.011)	0.8643 (0.003)	0.8241 (0.004)	0.9097 (0.002)
LR^g^	0.8551 (0.009)	0.8171 (0.005)	0.8259 (0.002)	0.8361 (0.003)	0.9089 (0.004)
SVM^h^	0.8370 (0.003)	0.8456 (0.002)	0.8390 (0.004)	0.8413 (0.009)	0.9172 (0.004)

^a^AUROC: area under receiver operating characteristic.

^b^XGBoost: extreme gradient boosting.

^c^AdaBoost: adaptive boosting.

^d^GBM: gradient boosting machine.

^e^LGBM: light gradient boosting machine.

^f^RF: random forest.

^g^LR: logistic regression.

^h^SVM: support vector machine.

### K-Fold Cross-Validation Results

**Figure 3:**
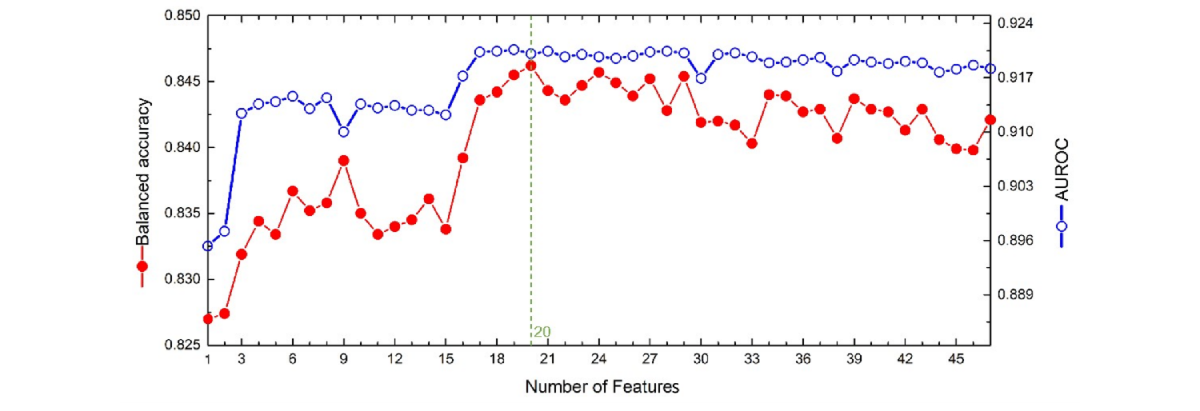
The influence of each top n-feature set on cross-validation. AUROC: area under receiver operating characteristic.

We evaluated the values of balanced accuracy and AUROC based on our 5-layer DNN according to the top n-features, where n={1, 2, …, 47}. The influence of each top n-feature set on AUROC and balanced accuracy is shown in Figure 3. According to the results, the values for both balanced accuracy and AUROC were saturated when the number of features for the input increased to more than 20. The top 20 features in our 5-layer DNN were selected from the results, yielding a sensitivity of 83.60%, specificity of 85.63%, accuracy of 84.07%, balanced accuracy of 84.62%, and AUROC of 0.9201. Note that similar accuracy metrics were obtained when all 47 features were used: sensitivity of 85.47%, specificity of 82.96%, accuracy of 84.88%, balanced accuracy of 84.21%, and AUROC of 0.9182. A summary of the comparison results is shown in Table 4. Also note that clinicians provisionally diagnosed all VLBWIs with RDS and provided them with artificial pulmonary surfactant treatment. We have included the prediction performance in Table 4. According to the results, both models including the top 20 ranked features (Table 5) and all 47 features showed similar prediction performance. Therefore, considering the complexity of the model, our 5-layer DNN including the top 20 features can be regarded as a better solution. In addition, note that the DNN shows higher accuracy in RDS prediction than SVM and other machine learning models, as shown in Tables 3 and 4.

**Table 4:** Cross-validation results of deep neural network (mean [SD]) with selected features.

Model	Sensitivity	Specificity	Accuracy	Balanced accuracy	AUROC^a^
Provisional diagnosis (clinicians)	1.000	0.000	0.7675	0.5000	N/A^b^
DNN^c^ with all 47 features	0.8547 (0.010)	0.8296 (0.016)	0.8488 (0.005)	0.8421 (0.004)	0.9182 (0.005)
DNN with the top 20 features	0.8360 (0.011)	0.8563 (0.010)	0.8407 (0.008)	0.8462 (0.005)	0.9201 (0.008)

^a^AUROC: area under receiver operating characteristic.

^b^N/A: not applicable.

^c^DNN: deep neural network.

**Table 5:** The top 20 ranked features in the models.

Feature rank	Feature name
1	Gestational age
2	Blood gas analysis within first hour of life
3	5-min Apgar score
4	Blood gas analysis base excess within first hour of life
5	Fourth order or more of multiple gestation
6	Body temperature at the initial admission
7	Third order of multiple gestation
8	Birth head circumference
9	Second order of multiple gestation
10	Medication usage at the initial resuscitation
11	Singleton
12	First order of multiple gestation
13	Marital status (divorced)
14	Birthplace not a hospital
15	Marital status (single)
16	Resuscitation at birth
17	Positive pressure ventilation usage at the initial resuscitation
18	Triplet gestation
19	Chronic hypertension
20	Birth weight

### Testing Data Results

An ensemble approach combining the 5 models derived from the 5-fold cross-validation was used for the final DNN model. Testing of our 5-layer DNN based on the ensemble approach was performed using the isolated testing data set (n=2618). A summary of the testing data results from comparison is shown in Table 6.

First, according to the results, our ensemble-based DNN model also showed accurate prediction performance on the isolated testing data: specificity of 86.78%, sensitivity of 83.24%, accuracy of 84%, balanced accuracy of 85.01%, and AUC of 0.9216. A comparison with the 5-fold cross-validation showed similar accuracy metrics, indicating that overfitting or underfitting was minimal. Indeed, slightly higher accuracy metrics were obtained since *p_m_* was combined from multiple models using 5-fold cross-validation by applying the weighted value of *A_m_* to *p_m_*. The highest values for both balanced accuracy and AUROC were obtained using our ensemble-based 5-layer DNN using the top 20 features, followed by ensemble-based DNN using all 47 features and all machine learning models. The cross-validation and the comparison showed similar prediction performances. Also note that clinicians provisionally diagnosed all VLBWIs with RDS and provided them with artificial pulmonary surfactant treatment. We have included the prediction performance in Table 6.

**Table 6:** Testing data results.

Model	Sensitivity	Specificity	Accuracy	Balanced accuracy	AUROC^a^
Ensemble-based DNN^b^ with top 20 features	0.8324	0.8678	0.8400	0.8501	0.9216
Ensemble-based DNN with all 47 features	0.8160	0.8792	0.8307	0.8476	0.9211
DNN single model with top 20 features	0.8313	0.8662	0.8394	0.8488	0.9198
DNN single model with all 47 features	0.8345	0.8559	0.8395	0.8452	0.9184
SVM^c^	0.8359	0.8564	0.8407	0.8461	0.9204
LR^d^	0.8398	0.8482	0.8418	0.8440	0.9208
RF^e^	0.8463	0.8401	0.8448	0.8432	0.9187
LGBM^f^	0.8359	0.8531	0.8399	0.8445	0.9178
GBM^g^	0.8329	0.8548	0.8380	0.8438	0.9073
AdaBoost^h^	0.8393	0.8417	0.8399	0.8405	0.9169
XGBoost^i^	0.8373	0.8482	0.8399	0.8428	0.9172
Provisional diagnosis (clinicians)	1.000	0.000	0.7673	0.5000	N/A^j^

^a^AUROC: area under receiver operating characteristic.

^b^DNN: deep neural network.

^c^SVM: support vector machine.

^d^LR: logistic regression.

^e^RF: random forest.

^f^LGBM: light gradient boosting machine.

^g^GBM: gradient boosting machine.

^h^AdaBoost: adaptive boosting.

^i^XGBoost: extreme gradient boosting.

^j^N/A: not applicable.

### RDS Incidence Rates

RDS, a significant disease affecting premature infants [42], is caused by a deficiency in the amount of pulmonary surfactant [43]. It is attributed to the presence of immature type 2 pneumocytes in the pulmonary alveoli; therefore, RDS incidence is greater with lower gestational age [42]. According to the KNN database utilized in this study, the incidence of RDS was 39.2% (302/770) at 32 weeks, 69% (1044/1513) at 30 weeks, 92.51% (1557/1683) at 28 weeks, and 98.1% (953/971) at 25 weeks. A summary of the incidence rates of RDS according to the gestational weeks is shown in Table 7.

**Table 7:** Respiratory distress syndrome occurrence rate.

Gestational week	Non–respiratory distress syndrome (n=3046), n	Respiratory distress syndrome (n=10,041), n	Total (N=13,087), n	Respiratory distress syndrome rate (%) (total=76.72%)
22	3	124	127	97.63
23	7	457	464	98.49
24	15	740	755	98.01
25	18	953	971	98.14
26	34	1160	1194	97.15
27	72	1301	1373	94.75
28	126	1557	1683	92.51
29	245	1550	1795	86.35
30	469	1044	1513	69
31	497	619	1116	55.46
32	468	302	770	39.22
33	413	125	538	23.23
34	370	66	436	15.13
35	222	32	254	12.59
36	87	11	98	11.22

### AI-Driven Web Application

**Figure 4:**
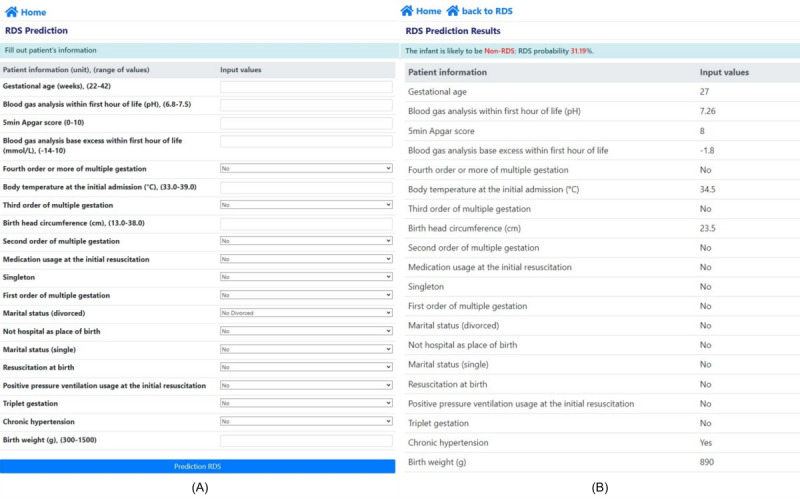
Deployed artificial intelligence–driven web application. (A) Web application before uploading patients’ information. (B) Web application after uploading patients’ information. RDS: respiratory distress syndrome.

Our proposed AI model for the prediction of RDS in VLBWIs was successfully deployed on our own public website [23]. The deployed web application, which provides results for the prediction of RDS, is shown in Figure 4. The web interface for entering information on the 20 features of VLBWI by a user is shown in Figure 4A. After entering the information in the web application, a user can immediately obtain the results for the prediction of RDS, as shown in Figure 4B. The prediction results also include the probability of RDS.

## Discussion

### Principal Findings

Once a timely diagnosis has been made after birth, treatment entails administration of artificial pulmonary surfactant through the trachea [44]. Survival of extremely premature infants has shown recent improvement, and various methods have been adopted for the administration of surfactant. The surfactant is usually administered through an intubated endotracheal tube, followed by administration of mechanical ventilation. The use of the intubation-surfactant-extubation method involves early extubation of the endotracheal tube, immediately after the administration of the surfactant, in order to avoid the use of consecutive mechanical ventilation and unnecessary positive pressure [45]. A steady increase in the use of LISA among practices has recently been reported [46]. Although LISA does not require the use of an intubation tube with a large bore, it requires the placement of a thin catheter in the trachea for the instillation of surfactant in patients who demonstrate self-respiration in noninvasive ventilation such as nasal continuous positive airway pressure. Regarding the problem of who should receive treatment or when treatment should be administered, wide, uniform administration of prophylactic treatment in premature infants younger than a certain gestational age such as 30 weeks is considered customary [47]. This policy does not consider premature infants who do not have RDS. Early rescue therapy has been established as one of the guidelines, meaning that the selection of patients should be based on worsening of the condition under a certain level of oxygen support and continuous positive airway pressure [6]. These various methods have been developed as a result of the effort to administer treatment for RDS while avoiding unnecessary trauma resulting from positive pressure caused by the procedure [6]. However, the decision to differentiate non-RDS infants in advance is not concrete, particularly in the hustle and bustle of the delivery room, because physicians working in such settings are prone to act in haste, with administration of surfactants and concluding the procedure. Therefore, the use of our AI-driven predicting tool would be helpful in the effort to make more prudent decisions.

According to the data on 13,087 infants weighing less than 1500 grams who were included in our study, RDS was diagnosed in 10,041 (76.72%) infants; however, 3046 (23.28%) infants did not have RDS, indicating that the surfactant was administered unnecessarily in 23.28% (3046/13,087) of the infants who did not have RDS, and these infants suffered from barotrauma as well. Prenatal and perinatal information were selected from the KNN database for use as input data for our AI model so that the tool for prediction of RDS was derived from machine learning and DNN models. The web application of our AI model can enable the preparation of more stable administration of resuscitation. Our AI-driven tool is open on [23]. We believe that allowing public access to the AI model will facilitate the validation and improvement of the model.

### Limitations and Future Works

Regarding the overfitting issue, we confirmed that the issue was minimal because the cross-validation results and the isolated testing data results were almost overlapping. Nevertheless, the potential overfitting issue still remained. First of all, as summarized in Table 6, the gestational age of the VLBWIs was generally distributed between 26 and 32 weeks. Therefore, our developed model may be biased toward the gestational week range of 26 to 32 weeks. Indeed, our recent study showed that data skewed toward 1 gender can also lead to model bias [48]. Thus, in the future work, we need to update our model based on a more balanced gestational week data.

Multiple gestations, maternal age, nulliparous, assisted reproductive technology, mode of delivery, gender of newborn, and birth weight are well-known risk factors for RDS [49]. Considering the risk factors, integrated scoring was mainly used to determine the risk for infants. Our AI tool used 47 input data sets encompassing most of the known risk factors for RDS. As shown in Figure 3, a border was observed until the 20th input factor rendered maximum power and was attenuated thereafter. The gestational age was confirmed as the most powerful factor, as shown in Figure 2. Of particular interest, new findings were observed on the graph of feature importance, rather than classical risk factors such as antenatal steroid (24th highest contributor), type of delivery (26th highest contributor), premature rupture of membrane (29th highest contributor), maternal diabetes (30th highest contributor), and birth weight (20th highest contributor). Interestingly, our model used the top 20 features, which do not contain most of the risk factors mentioned above: only the birth weight was used in our final model input with the least contribution. Nevertheless, we have not considered the effects of asphyxia, second-born twin, chronic intrauterine growth restriction, and prolonged labor, which are also well-known risk factors. In addition, genetic information, environmental data, maternal nutritional status, and smoking/alcohol habits may also influence the development of RDS. We believe that those factors can make an important contribution to RDS prediction in VLBWIs. Thus, in the future work, we plan to suggest that the KNN registry adds additional risk factors and update our prediction model.

Furthermore, we will use our developed web application to acquire additional data and validate the model. Currently, the deployed application does not store any information entered by users to protect personal data. However, we plan to store information entered by the users upon agreement to improve the AI model via a real-time learning process. Last, in this study, we did not consider postnatal factors because we aimed to develop an AI model that predicts the outcome of VLBWIs at the time of their birth. In the future work, we will also consider postnatal factors to enhance the prediction performance.

### Conclusions

The strength of our study is that it utilized the first AI-driven model for the prediction of RDS in VLBWIs, based on quality data from the KNN. The Korean Disease Control and Prevention Agency provides funding and supervision for the KNN registry; therefore, the data are well-qualified. The sample size of 13,087 is large, considering the weight of national data on VLBWIs. The guidelines for the management of RDS have been established, and the relatively large amount of data supports the strength of our study. Our AI tool is open to the public on the website for application by physicians in the clinical setting. In conclusion, our AI-driven tool for the prediction of RDS showed balanced accuracy of 85.26% and AUROC of 0.9187. Because our AI-driven tool can be used in the prediction of RDS, neonatal teams will find it helpful in the preparation of neonatal resuscitation, including instillation of surfactant in the setting of VLBWI delivery. We suggest to conduct further research to expand to international cohorts, including diverse racial backgrounds, to generalize our AI model.

## Appendix

Multimedia Appendix 1 The results of feature importance analysis of the other 6 machine learning models: extreme gradient boosting, adaptive boosting model, gradient boost machine, light gradient boosting machine, random forest, and logistic regression.

## Abbreviations

AdaBoost: adaptive boosting
AI: artificial intelligence
AUROC: area under receiver operating characteristic
DNN: deep neural network
GBM: gradient boosting machine
KNN: Korean neonatal network
LGBM: light gradient boosting machine
LISA: less invasive surfactant administration
LR: logistic regression
RDS: respiratory distress syndrome
RF: random forest
SVM: support vector machine
VLBWI: very low birth weight infant
XGBoost: extreme gradient boosting

## Data Availability

The data are not publicly available due to restrictions from the Korean Neonatal Network policy, as they belong to the Korean Neonatal Network. Details of accessing the raw data and guide are available at [50].
