# Supplementary material for: Artificial Intelligence–Driven Respiratory Distress Syndrome Prediction for Very Low Birth Weight Infants: Korean Multicenter Prospective Cohort Study
Source: J Med Internet Res. 2023 Jul 10;25:e47612. doi: 10.2196/47612 (PMC10366668; doi:10.2196/47612)
Supplement: Multimedia Appendix 1 [file jmir_v25i1e47612_app1.docx]

**Supplementary data**





Figure S1. Results of feature importance analysis from XGBoost





Figure S2. Results of feature importance analysis from Adaboost model.





Figure S3. Results of feature importance analysis from gradient boost model





Figure S4. Results of feature importance analysis from light gradient boost model





Figure S5. Results of feature importance analysis from random forest





Figure S6. Results of feature importance analysis from logistic regression.
